# Injury of the Head—Abscess, with Absence of Symptoms, Etc.

**Published:** 1870-12

**Authors:** C. T. Parkes

**Affiliations:** Demonstrator of Anatomy in Rush Medical College


### Chicago Medical Journal.

A MONTHLY RECORD OF

*Medicine, Surgery, and the Collateral Sciences.*

EDITED BY J. ADAMS ALLEN, M.D., LL.D.; AND WALTER HAY, M.D.

VOL. XXVII.—DECEMBER, 1870.—NO. 12.

### Original Communications.

ARTICLE I. —*Injury of the Head — Abscess, with absence of Symptoms — Trepanning — Post Mortem Examination.* By C. T. PARKES, M.D., Demonstrator of Anatomy in Rush Medical College.

On October 8th, 1870, was called to see Mr. T. Q——, who, two hours previous to my arrival, had been struck on the head with a stone of some kind, in a quarrel. The blow knocked him down. Did not gain any history of unconsciousness. He was perfectly sensible when I reached him; had no pain or dizziness in the head; expressed himself as greatly concerned about the shame of the quarrel.

Examination showed me a contused wound just external to left frontal eminence, extending through the scalp, and about an inch and a quarter long. Also, a clean cut, T shaped, wound, at the upper and back part of the left parietal bone, extending through scalp and pericranium, completely denuding the bone; long arm of cut an inch and a half long, and short arm half an inch longer.

The man was bleeding per saltum from the latter wound, and had been bleeding thus ever since receiving his injuries. By placing a finger upon the divided artery of the wound and holding it a few minutes, all hæmorrhage ceased; upon changing his position to dress the wounds, the man fainted, showing that his loss of blood had been very severe. He soon rallied; I then examined the skull thoroughly for fracture or depression, and found neither. The wounds were closed by sutures — two in the wound on the back of the head, and one in the cut on the front; water and whisky dressings, to be applied tepid.

The man put on his hat and walked home, feeling, as he expressed it, " all right."

Oct. 9th. Feels well, and wounds looking nicely; pulse 72, no headache.

Oct. 10th. Wound on back of head looks angry; a pretty well marked puffy tumor surrounding it. Took out the sutures, allowing about a teaspoonful of healthy pus to ooze from the wound; ordered the application of a good warm poultice.

The wound on the forehead still looking well; seems to be union by first intention; complains of a little headache; pulse 80. Gave two compound cathartic pills; ordered to keep in bed.

Oct. 11th. Wound on back of head still quite angry and inflamed; the surrounding puffiness increased. Broke up all slight adhesions in the wound, and gave exit to considerable quantity of pus; continue poultice. Wound on front of head swollen, puffy, and painful; took out the suture; broke up the adhesions slightly with the probe, and got pus; ordered poultice to this wound. Found every little scratch and abrasion on the man's scalp suppurating and painful; opened all the little pockets; headache increased; bowels freely moved; pulse 84. Directed light diet and quietude.

Oct. 12th. Man better, decidedly; pulse 76; headache gone; wounds suppurating slightly, and beginning to look healthy; puffiness gone; probed the wound at back of the head, and thought I got the ringing sound, as from " necrosed " bone.

Treatment continued.

13th and 14th. Doing well; appetite good.

15th. Doing well; discontinued poultices, and applied ointment of Carbolic Acid Cryst. gr. v, to ℥j Resin Cerate, spread on Oakum, to the wounds.

During next week seemed to be doing quite well; appetite was not very good; vomited his food once or twice; attributed his want of appetite to the confinement upon which I insisted for fear of brain trouble; he had always been an active, hard working man. The wound on forehead healed up entirely; that on back of the head was healing rapidly towards the centre of the crossed cut. Satisfied that he had quite a piece of "necrosed" bone at the bottom of wound. The pulse during this week never passed beyond 80 beats per minute.

Oct. 25th. Patient thought the "dead bone" should be removed; advised him to wait until nature loosened it. Took him to see Professor Gunn, of Rush Medical College. He confirmed the diagnosis; advised the patient to await the efforts of nature at separation; to make use of a more generous diet, and to take a little more outdoor exercise.

From this until October 28th, noticed no adverse change in the patient; did not complain so much of nausea; in fact, said he "didn't feel sick." The wound on the back of the head had healed, all but an opening sufficiently large to admit the probe. Bowels freely open.

On the 28th of October found him anxious and worse; pulse 84, and a feeling of numbness in right arm, the side opposite to site of injury. Told him to keep perfectly quiet, and that I would bring Dr. M. Gunn to see him early in the morning.

Oct. 29th. Dr. Gunn and myself saw the man at 9 A. M.; found him speechless, with laborious breathing, and paralysis of the side opposite to the seat of injury; pulse 108, and jerking; evident symptoms of compression. Dr. Gunn trephined at the site of the wound on back part of the head. On the removal of the outer table of the skull, we found quite a quantity of pus in the "diploe"; under the inner table there was a thick layer of lymph covering the dura mater. The dura mater did not bulge into the trephine opening; the pulsations of the brain were visible; the lymph showed several specks of degeneration into pus.

After the operation the man's countenance looked brighter; his pulse fell to 84, and in answer to the question if he was better, he said, "I am"; improved by the operation. In the afternoon found him no better; all the morning symptoms present. He gradually continued to grow worse until the morning of Nov. 1st, when he died—three weeks and three days after receiving his injury.

Post mortem seven hours after death made by Professor Moses Gunn, Dr. Ben. C. Miller, county physician, and myself. On removing the scalp, no evidences of either fracture or depression were visible on the outside of the calvarium. On removal of the calvarium, no evidence whatever of any injury to the inner table of the same could be found. There was a slight deposit of lymph on the dura mater at the site of injury. The vessels of the membrane in this neighborhood slightly congested; no other evidence of meningeal trouble present. On cutting open the dura mater, found the arachnoidian cavity free from fluid, and the parietal and visceral layers of the arachnoid membrane bright and glistening, seemingly perfectly healthy. Beneath the visceral layer of the arachnoid membrane, on the surface of the cerebral mass, we found a well defined layer of yellow lymph, spread over the posterior three-fourths of the left cerebrum, and extending slightly on the anterior portion of the right hemisphere. The lymph was oldest and thickest immediately beneath the site of injury. Here it was about one-fourth of an inch thick, and left a smooth surface upon being cut into.

The substance of the brain presented nothing noticeably abnormal. Upon opening the lateral ventricles, each discharged at least one ounce of serous purulent fluid.

During the whole course of his illness this man never showed any symptoms which pointed positively to trouble with the brain, until October 28th, unless the want of appetite was a symptom indicating it. He complained of no headache, except four or five days after injury, when all the wounds took upon themselves unhealthy action. When they improved, the headache departed. At this time, when suppuration was at its height, the pulse rose to 84 beats per minute; at all other times it was below 80, until the 28th, when it came up to 84 again. On the 29th—day of operation, 108; day of death, 120.

The man was kept as quiet as possible during the whole time, without being absolutely in bed. The case is surely instructive in indicating a guarded prognosis even in seemingly trivial injuries to the head.

